# Effects of Short-Term Probiotic Ingestion on Immune Profiles and Microbial Translocation among HIV-1-Infected Vietnamese Children

**DOI:** 10.3390/ijms18102185

**Published:** 2017-10-19

**Authors:** Azumi Ishizaki, Xiuqiong Bi, Lam Van Nguyen, Kazunori Matsuda, Hung Viet Pham, Chung Thi Thu Phan, Dung Thi Khanh Khu, Hiroshi Ichimura

**Affiliations:** 1 Department of Viral infection and International Health, Graduate School of Medical Sciences, Kanazawa University, Kanazawa 920-8640, Japan; aishizak@staff.kanazawa-u.ac.jp (A.I.); bixiuqio@staff.kanazawa-u.ac.jp (X.B.); 2 National Hospital of Pediatrics, Hanoi 100000, Vietnam; dinhlam73@yahoo.com (L.V.N.); vhnhi44@gmail.com (H.V.P.); phanthuchung@gmail.com (C.T.T.P.); hangdung2001@yahoo.com (D.T.K.K.); 3 Yakult Central Institute, Tokyo 186-8650, Japan; kazunori.matsuda@yher.be

**Keywords:** HIV-infected children, probiotics, intestinal microbial translocation, immune activation, 16S/23S ribosomal DNA

## Abstract

Here, we investigated the effects of the probiotic strain *Lactobacillus casei* Shirota (LcS) on immune profiles and intestinal microbial translocation among children infected with human immunodeficiency virus (HIV). This prospective study included 60 HIV-infected children—including 31 without antiretroviral therapy (ART) (HIV(+)) and 29 who received ART for a median of 3.5 years (ART(+)) and 20 children without HIV infection (HIV(−)). Participants were recruited in Vietnam. All children were given fermented milk containing LcS (6.5 × 10^9^ cfu) daily for 8 weeks. Before and after LcS ingestion, blood samples were collected for virological, immunological, and bacteriological analyses. After LcS ingestion, peripheral CD4^+^ T-cell and Th2 (CXCR3^−^CCR6^−^CD4^+^) counts significantly increased in both HIV-infected groups; Th17 (CXCR3^−^CCR6^+^CD4^+^) counts increased in all three groups; regulatory T-cell (CD25^high^CD4^+^) counts decreased in the ART(+) and HIV(−) groups; activated CD8^+^ cells (CD38^+^HLA-DR^+^CD8^+^) decreased from 27.5% to 13.2% (*p* < 0.001) in HIV(+) children; and plasma HIV load decreased slightly but significantly among HIV(+) children. No group showed a significantly altered frequency of bacterial 16S/23S rRNA gene detection in the plasma. No serious adverse events occurred. These findings suggest that short-term LcS ingestion is a safe supportive approach with immunological and virological benefits in HIV-infected children.

## 1. Introduction

Human immunodeficiency virus type 1 (HIV) mainly infects CD4^+^ cells, and the gut-associated lymphoid tissue (GALT) is the largest HIV replication site and reservoir [1,2]. Early-stage HIV infection involves remarkable destruction of CD4^+^ T cells in the GALT, particularly in the Th17 subset [1,2,3]. This promotes a decline in the immune and mechanical barrier functions of the gut mucosa, and the subsequent translocation of microbial products (e.g., bacterial DNA) from the gastrointestinal tract to systemic circulation [4,5]. It has been proposed that intestinal microbial translocation is associated with chronic systemic immune activation [6], further disruption of immune balance, and loss of systemic CD4^+^ T cells [4]. Antiretroviral therapy (ART) can successfully suppress HIV replication and induce recovery of CD4^+^ T cells in the peripheral blood in 1–3 years. However, ART-induced recovery of CD4^+^ T cells is much slower in the GALT than in peripheral blood, and the gut mucosal barrier function remains impaired even after a long period of ART [1,2,7,8]. Failure to restore Th17 cells in the GALT may impair both the recovery of gut mucosal barrier function and the clearance of microbial products [2], suggesting the need for novel therapeutic approaches to restore Th17 cells in the GALT of patients with HIV [2].

Probiotics and prebiotics reportedly reinforce mucosal barrier function and modulate immune responses by affecting the intestinal epithelium and GALT [9]. Prior studies demonstrate the safety of administering probiotics/prebiotics to HIV-infected individuals. Oral administration of probiotics/prebiotics in this population reportedly increases peripheral CD4^+^ T-cell counts, reduces CD4^+^ T-cell activation, changes the intestinal bacterial flora, and decreases the plasma concentrations of bacterial DNA and soluble CD14 (sCD14) [10,11,12,13,14].

The probiotic strain *Lactobacillus casei* Shirota (LcS) has been used to make fermented dairy products for over 80 years in Japan and many other countries. LcS is a multifunctional probiotic that acts as an immune-stimulant and an immune-regulator [15]. Moreover, it is reportedly associated with increased T lymphocytes and significantly decreased mRNA levels of TGFβ, IL10, IL12, and IL-1β in HIV-infected adults who received ART [16].

We recently reported that HIV infection in Vietnamese children causes a rapid reduction of Treg cells; slow decreases of Th1, Th2, and Th17 cells; and early CD8^+^ cell activation. Moreover, we showed that ART in this population induces rapid Th1 recovery and suppresses CD8^+^ cell activation, while the normalization of Th2/Treg/Th17 cells requires 5–8 years of ART [17]. In our current study, we examined the same population of Vietnamese children with HIV infection, with the aim of elucidating how the probiotic LcS influenced the immune profile and bacterial translocation.

## 2. Results

This prospective study included 60 HIV-infected children—including 31 without ART (HIV(+)) and 29 who had received ART for a median of 3.5 years (range: 0.8–5.8 years) (ART(+)). The study also included 20 children without HIV infection (HIV(−)). All participants were recruited in Vietnam, as previously reported [17]. All children were given fermented milk containing LcS (6.5 × 10^9^ cfu) daily for 8 weeks.

### 2.1. LcS Ingestion Did Not Induce Significant Clinical Events

After LcS ingestion, three potentially LcS-related clinical events were observed in the HIV(+) group. Two children had watery stool starting on the day after LcS initiation and lasting for a few days. One child had rashes covering the whole body on the day after LcS initiation and lasting for 3 days. All children continued LcS ingestion and all symptoms resolved without any treatment. No clinical events related to LcS ingestion were observed in the ART(+) and HIV(−) groups.

Several clinical events that were not clearly related to LcS ingestion occurred during the study period. Ten such events occurred among seven HIV(+) children, including upper respiratory infection symptoms in four children, intestinal infection symptoms in two children, loss of >5% of body weight in two children, fever in one child, and tonsillitis and skin rashes in one child. Six clinical events occurred among three ART(+) children, including upper respiratory infection symptoms in two children, chronic purulent ear infections in two children (both of whom had symptoms before LcS ingestion), the recurrence of a chronic purulent ear infection in one child, and loss of >5% of body weight in one child. No clinical events were observed in the HIV(−) children during the study period.

### 2.2. LcS Ingestion Improved Physical and Clinical Parameters

**Figure 1:**
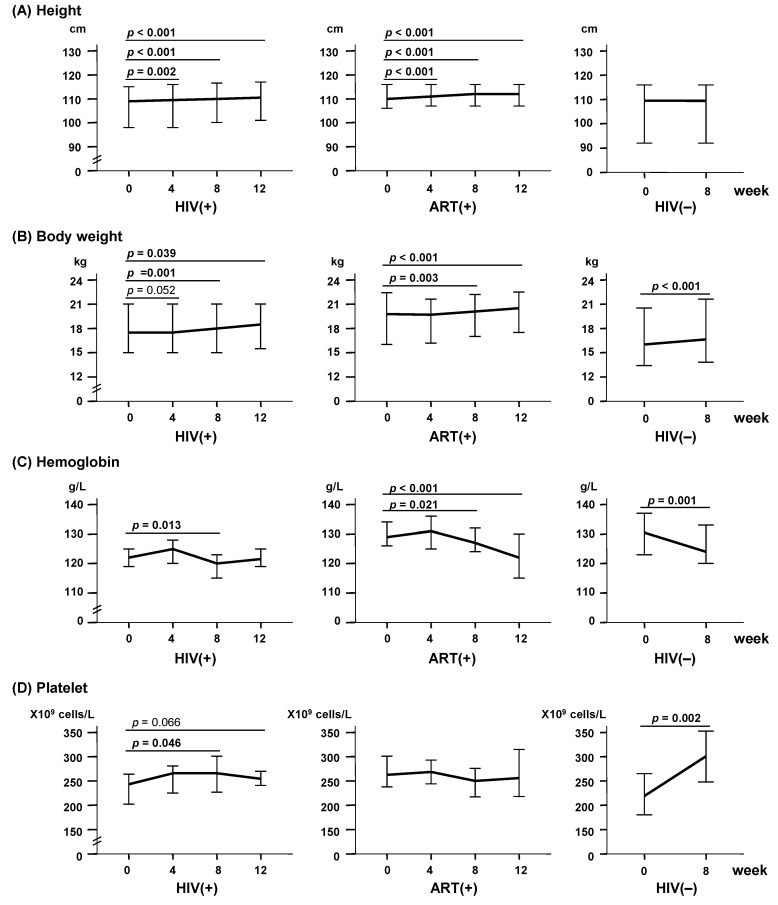

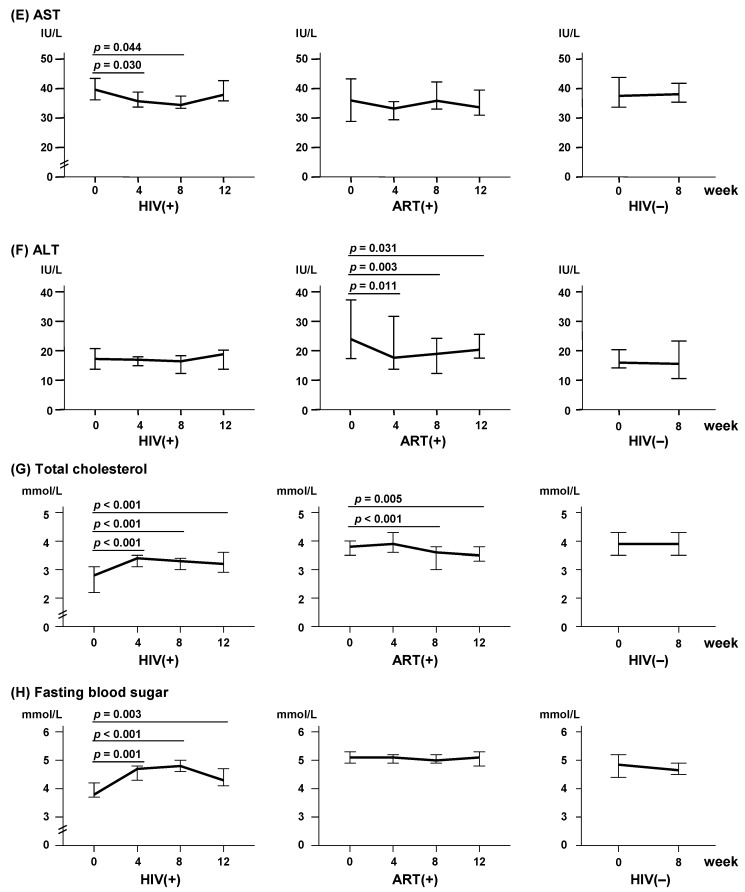
Changes of physical and clinical parameters after *Lactobacillus casei* Shirota (LcS) ingestion in the HIV(+) (*n* = 31), ART(+) (*n* = 29), and HIV(−) (*n* = 20) groups. (**A**) Height; (**B**) Body weight; (**C**) Hemoglobin; (**D**) Platelet counts; (**E**) Aspartate aminotransferase (AST; normal range: < 40 U/L); (**F**) Alanine aminotransferase (ALT; normal range: < 40 U/L); (**G**) Total cholesterol (normal range: 3.9–5.2 mmol/L); (**H**) Fasting blood sugar (normal range: 4.2–5.5 mmol/L). Bars represent the median and 95% confidential interval (CI) for each group. The *p* values show the comparison of weeks 4, 8, and 12 to week 0. HIV: Human immunodeficiency virus type 1; ART: Antiretroviral therapy.

After LcS ingestion, height significantly increased from week 4 to week 12 in both the HIV(+) and ART(+) groups (all *p* < 0.01), but not in the HIV(−) group (*p* = 0.317, Figure 1A). Body weight was significantly increased at week 8 in all three groups (*p* < 0.01, Figure 1B), and was further increased at week 12 in both the HIV(+) and ART(+) groups. All three groups showed slight but significant decreases in hemoglobin levels at week 8 (*p* < 0.05, Figure 1C). Platelet counts were significantly increased at week 8 in the HIV(+) and HIV(−) groups (*p* = 0.046 and 0.002, respectively; Figure 1D). With regard to liver function, aspartate aminotransferase (AST) levels significantly decreased from week 4 to week 8 in the HIV(+) group (*p* < 0.05, Figure 1E), and alanine aminotransferase (ALT) levels significantly decreased from week 4 to week 12 in the ART(+) group (*p* < 0.05, Figure 1F). Total cholesterol level significantly increased from week 4 to week 12 in the HIV(+) group (*p* < 0.001), but decreased from week 8 to week 12 in the ART(+) group (Figure 1G, *p* < 0.01). Fasting blood sugar level significantly increased from week 4 to week 12 in the HIV(+) group (*p* < 0.01), but did not significantly change in the ART(+) and HIV(−) groups (Figure 1H).

### 2.3. LcS Ingestion Increased CD4^+^ Cells, Especially Th2 and Th17 Subsets

**Figure 2:**
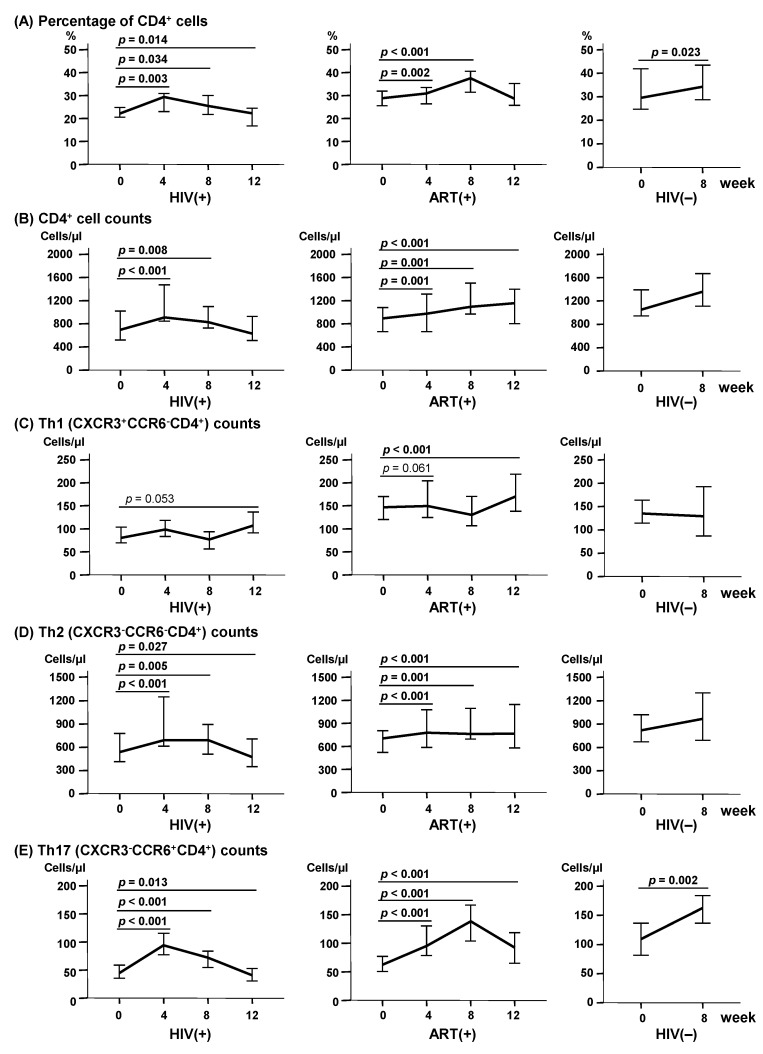

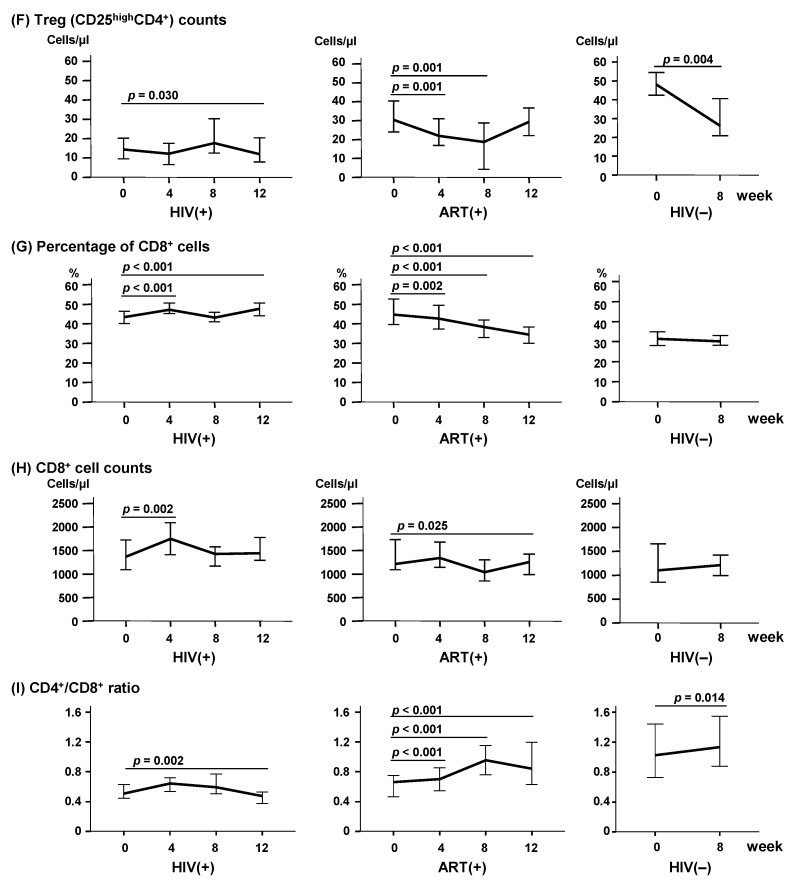
Changes of the CD4^+^ cell and subset counts and CD8^+^ cell counts after LcS ingestion in the HIV(+) (*n* = 31), ART(+) (*n* = 29), and HIV(−) (*n* = 20) groups. (**A**) CD4^+^ cell percentages among lymphocytes; (**B**) CD4^+^ cell counts; (**C**) Type 1 helper T cell, CXCR3^+^CCR6^−^CD4^+^ (Th1) counts; (**D**) Th2 (CXCR3^−^CCR6^−^CD4^+^) counts; (**E**) Th17 (CXCR3^−^CCR6^+^CD4^+^) counts; (**F**) Treg (CD25^high^ CD4^+^) counts; (**G**) CD8^+^ cell percentages among lymphocytes; (**H**) CD8^+^ cell counts; (**I**) CD4/CD8 ratio. Bars represent the median and 95% CI for each group. The *p* values show the comparison of weeks 4, 8, and 12 to week 0.

All three groups showed a significant increase of the CD4^+^ cell percentage among lymphocytes at weeks 4 and 8 during the LcS ingestion period (Figure 2A, *p* < 0.05). CD4^+^ cell counts also significantly increased in the HIV(+) and ART(+) groups (Figure 2B, *p* < 0.01), showing median increases of 213 cells/µL at week 4 and 131 cells/µL at week 8 in the HIV(+) group, and of 78, 199, and 260 cells/µL at weeks 4, 8, and 12, respectively, in the ART(+) group. Th1 (CXCR3^+^CCR6^−^CD4^+^) subset counts were significantly increased at week 12 in the ART(+) group (*p* < 0.001), and were marginally increased at week 12 in the HIV(+) group (*p* = 0.053, Figure 2C). In the HIV(+) group, Th2 (CXCR3^−^CCR6^−^CD4^+^) subset counts increased by a median of 154 cells/µL at week 4 (*p* < 0.001) and 158 cells/µL at week 8 (*p* < 0.005), and decreased by a median of 66 cells/µL at week 12 (*p* = 0.027). On the other hand, Th2 (CXCR3^−^CCR6^−^CD4^+^) subset counts were increased by medians of 80, 208, and 254 cells/µL at weeks 4, 8, and 12, respectively, in the ART(+) group (*p* ≤ 0.001); and did not significantly change in the HIV(−) group (Figure 2D). In all three groups, Th17 (CXCR3^−^CCR6^+^CD4^+^) subset counts were significantly increased at weeks 4 and 8 (*p* < 0.05, Figure 2E). In the HIV(+) group, Th17 cells increased by a median of 49 and 27 cells/µL at week 4 and week 8, respectively, and decreased by 4 cells/µL at week 12. In the ART(+) group, Th17 cells increased by 35, 80, and 22 cells/µL at weeks 4, 8, and 12, respectively. Th17 cells were increased by 53 cells/µL at week 8 in the HIV(−) group. In the ART(+) group, Treg subset counts decreased by a median of 8 and 11 cells/µL (both *p* = 0.001) at week 4 and week 8, respectively. Treg subset counts also decreased by 2 cells/µL at week 12 in the HIV(+) group (*p* = 0.030), and by 22 cells/µL at week 8 in the HIV(−) group (*p* = 0.004) (Figure 2F).

The CD8^+^ cell percentages among lymphocytes were significantly increased at weeks 4 and 12 in the HIV(+) group (*p* < 0.001), were significantly decreased at weeks 4, 8, and 12 in the ART(+) group (*p* < 0.01), and did not significantly change in the HIV(−) group (Figure 2G). On the other hand, the CD8^+^ cell counts were significantly increased at week 4 in the HIV(+) group, and at week 12 in the ART(+) group (*p* < 0.05, Figure 2H). The CD4/CD8 ratio was significantly increased at weeks 4, 8, and 12 (*p* < 0.001 each) in the ART(+) group and at week 8 in the HIV(−) group (*p* = 0.014), but decreased at week 12 in the HIV(+) group (*p* = 0.002) (Figure 2I).

### 2.4. LcS Ingestion Induced a Dramatic Decline in CD8^+^ Cell Activation But Not in Monocyte Activation

**Figure 3:**
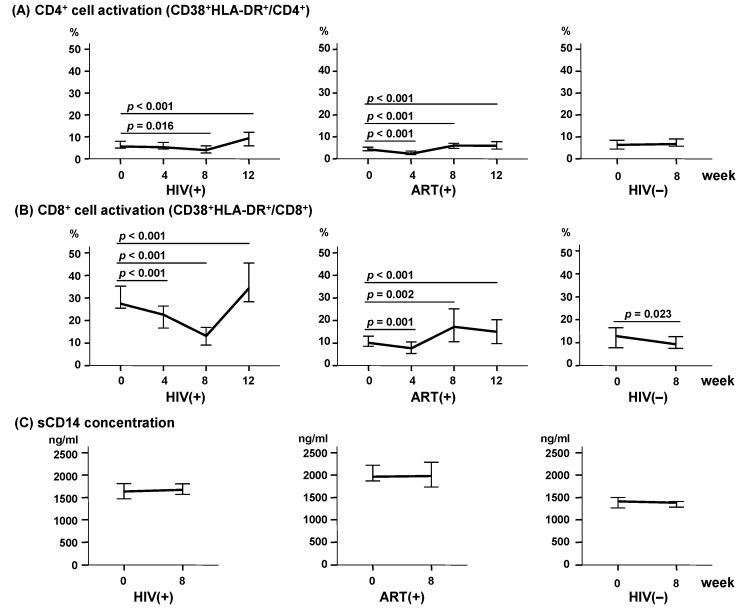
Change of cell activation after LcS ingestion in each study group. (**A**) Percentage of activated CD4^+^ cells (CD38^+^HLA-DR^+^/CD4^+^); (**B**) Percentage of activated CD8^+^ cells (CD38^+^HLA-DR^+^/CD8^+^); (**C**) Plasma sCD14 concentration (monocyte activation status). Bars represent the median and 95% confidence interval for each group. The *p* values show the comparison of weeks 4, 8, and 12 to week 0.

After LcS ingestion, the percentages of activated CD4^+^ cells (CD38^+^HLA-DR^+^CD4^+^) were slightly decreased at week 8 and were then increased at week 12 in the HIV(+) group (*p* = 0.016 and *p* < 0.001, respectively). These percentages were decreased at week 4 and then increased at weeks 8 and 12 in the ART(+) group (all *p* < 0.001), and did not significantly change in the HIV(−) group (Figure 3A). On the other hand, in the HIV(+) group, the percentages of activated CD8^+^ cells (CD38^+^HLA-DR^+^CD8^+^) significantly decreased from 27.5% at week 0 to 22.6% at week 4 (*p* < 0.001) and to 13.2% at week 8 (*p* < 0.001), but rebounded to 34.3% at week 12 (*p* < 0.001). In the ART(+) group, these percentages decreased from 10.2% at week 0 to 7.8% at week 4 (*p* = 0.001), and rebounded to 17.3% at week 8 (*p* = 0.008) and 15.0% at week 12 (*p* < 0.001). They also significantly decreased from 12.9% at week 0 to 9.3% at week 8 in the HIV(−) group (*p* = 0.023) (Figure 3B).

After LcS ingestion, the plasma sCD14 concentration (a marker of monocyte activation) was not significantly changed at week 8 in any of the three groups (*p* = 0.364–0.852, Figure 3C).

### 2.5. LcS Ingestion Induced a Decrease in Plasma Viral Load (VL) in HIV(+) Children

In the HIV(+) group, the plasma HIV VL decreased slightly but significantly from a median of 5.0 log_10_ copies/mL at week 0 to 4.7 log_10_ copies/mL at week 8 (*p* = 0.004). On the other hand, the VL did not significantly change in the ART(+) group (*p* = 0.878) (data not shown).

### 2.6. LcS Ingestion Did Not Significantly Change Microbial Translocation

Table 1 shows the detection of 12 representative gut bacterial 16S/23S rDNA in plasma samples before and after LcS ingestion. Bacterial rDNA derived from five bacteria—*Staphylococcus*, *Streptococcus*, *Pseudomonas*, *Enterobacteriaceae*, and *Prevotella*—were detected with frequencies of 0–30% in each group during the study period. The detection frequencies did not significantly change after LcS ingestion in any of the three groups (all *p* > 0.05).

We also analyzed bacterial 16S/23S rRNA molecules in the whole blood samples with RT-qPCR to evaluate the presence of live bacteria. In the whole blood samples collected from all children throughout the study period, we detected no bacterial rRNA molecules, with one exception. *Pseudomonas* rRNA was detected, with 15 cells/mL blood, in one child from the ART(+) group at week 8 of LcS ingestion (data not shown).

## 3. Discussion

In this study, we evaluated the effects of short-term (8 weeks) ingestion of fermented milk containing *Lactobacillus casei* strain Shirota (LcS) in children with HIV infections. Our results showed that LcS ingestion was safe for the HIV-infected children. Moreover, after LcS ingestion, the HIV-infected children (regardless of ART) showed significant increases of CD4^+^ cells, especially the Th17 subset, along with significant decreases in the percentages of activated CD8^+^ cells. Importantly, LcS ingestion was not associated with increased plasma HIV load or severe adverse events. These findings suggest that LcS ingestion may be a good supportive approach for HIV-infected children.

We found that peripheral CD4^+^ cell counts significantly increased in children of the HIV(+) group after LcS ingestion. This is consistent with the results of previous studies that have tested different kinds of probiotics in adults with HIV infection [9,10,11,13]. Analyses of CD4^+^ cell subsets revealed that LcS ingestion led to significant increases of Th17 subset counts in all three groups, although this increase was not maintained after LcS cessation. Th2 cell counts also increased in the HIV(+) and ART(+) groups, but not in the HIV(−) group. Th1 cell counts did not significantly change in any of the three groups. These results indicate that LcS ingestion was associated with increases of CD4^+^ cells, particularly the Th17 subset, in children with HIV infection. Supporting these results, one previous study reported that Th17 cell development was regulated by microbiota [18]. Another study found that LcS upregulated IL-17 production in draining lymph node lymphocytes in an experimental mouse model of encephalomyelitis [19]. These findings suggest that LcS may upregulate IL-17 production by increasing Th17-promoting antigen presenting cells in the gut, as observed in simian immunodeficiency virus (SIV)-infected macaques [20].

It has been previously reported that probiotics and prebiotics decrease the activation of CD4^+^ cells (CD4^+^/CD25^+^) without impacting the activation of CD8^+^ cells (CD38^+^HLA-DR^+^) [13]. However, recent reports have indicated that probiotics significantly reduced the activation of both CD4^+^ and CD8^+^ cells in HIV-infected adults receiving ART [21,22]. In our present study, LcS ingestion dramatically decreased CD8^+^ cell activation in the HIV(+) group to the level seen in the HIV(−) group, while CD8^+^ cell activation in the ART(+) group seemed to fluctuate within the range of the HIV(−) group. LcS ingestion tended to similarly change the activation of CD4^+^ and CD8^+^ cells, in both the HIV(+) and ART(+) groups, but with generally more modest changes in CD4^+^-cell activation. Shida et al. previously reported that LcS has both immunomodulatory and immunostimulatory functions, stimulating IL-12 production and also regulating IL-10 production in macrophages and dendritic cells located in the GALT [15]. Thus, our present findings suggest that LcS may adjust the imbalance of immune-cell activation in children with HIV infection, with or without ART.

Throughout our entire study period, no bacterial 16S/23S rRNA molecules were detected in the children’s blood, with one exception. *Pseudomonas* rRNA was detected once at week 8 in one child in the ART(+) group. This result indicates that live bacteria are rarely present in the blood of the children both with and without HIV infection. Although the bacterial 16S/23S rDNA was detected more frequently in plasma than rRNA molecules in blood, LcS ingestion did not lead to any significant change in the detection frequencies from any group. These findings suggest that short-term LcS ingestion does not significantly impact microbial translocation in children, regardless of HIV infection. Therefore, the significant decrease in CD8^+^ cell activation and the increase of CD4^+^ cells in the HIV-infected children was likely not primarily due to the inhibition of microbial translocation. Instead, these changes were probably caused by other factors associated with gut homeostasis, such as interactions between LcS and dendritic cells, regulatory T cells, innate lymphoid cells, other commensal bacteria, etc. [18]. Further studies are needed to clarify the mechanism.

The HIV(+) group showed slight but significant decreases in both plasma HIV VL after 8 weeks of LcS ingestion (*p* = 0.028) and in the activation of CD4^+^ cells, which are the main sites of HIV replication. This group also showed a significant increase in CD8^+^ cell percentages and counts at week 4, part of which may have an anti-viral function. Thus, the decreased plasma HIV VL could be associated with the decreased CD4^+^-cell activation and the increased CD8^+^ cells, and this is the first study to show the possibility that probiotics ingestion could induce a significant decrease in plasma VL. However, the median decrease in VL after LcS ingestion was only 0.3 log, which is far below the 1.5-log decrease required to define treatment success. We cannot exclude the possibility that the VL decrease might be due to variations in the quantitative VL assay or individual fluctuations in plasma VL. Further study is needed to clarify the possible function of probiotics such as LcS on HIV VL.

Total cholesterol, fasting blood sugar, height, and body weight were all lower in the HIV-infected groups than in the HIV(−) group at baseline, and all significantly increased in the HIV-infected groups after ingestion of LcS-containing fermented milk. These findings suggest that the LcS-supplemented fermented milk was beneficial in terms of improving the nutrition and growth of HIV-infected children, as reported previously [23]. On the other hand, all three groups showed significantly decreased hemoglobin levels after LcS ingestion, though the levels remained within the normal range. This finding may be consistent with previous results showing that 6 months of probiotic intake did not promote recovery from anemia in children without HIV infection [23]. A longitudinal study is needed to elucidate the influence of LcS intake on hemoglobin levels among children with HIV infection.

Baseline ALT levels were significantly higher in the ART(+) group than in the HIV(+) and HIV(−) groups, although all levels were within the normal range. After LcS ingestion, ALT and AST levels significantly decreased in both the ART(+) and HIV(+) groups. These findings suggest that LcS may have a protective effect on the liver. This is consistent with a recent report indicating that probiotics could limit inflammatory liver damage by modulating the intestinal flora, modifying gut mucosa functions, and decreasing the production of cytokines such as TNF-α [24,25].

Shortly after the initiation of LcS ingestion, three children in the HIV(+) group developed clinical symptoms that were likely related to LcS. The HIV(+) group also experienced more infectious events than the other groups during the study period, although these events were not directly related to LcS ingestion. All symptoms were resolved without any treatment and without LcS cessation. These findings suggest that LcS is clinically safe for ingestion by HIV-infected children.

This study had several limitations. First, we used cell surface markers to define the CD4^+^ cell subsets Th1, Th2, and Th17, instead of defining the subsets based on cytokine production (e.g., IFN-γ, IL-4, IL-17A) or transcription factors (e.g., FoxP3) because of the limited amount of blood samples (2–3 mL/child) collected from the children. Since Th2 cells were defined by CXCR3^−^CCR6^−^CD4^+^ expression, the Th2 group may have included other CD4^+^ subpopulations, such as Th22 cells, a new CD4^+^ subset described by Trifari et al. [26]. Therefore, we must be careful when considering a possible Th1/Th2 imbalance in HIV(+) groups, and in analyzing the changes observed after LcS ingestion. Second, the present results were based on only 8 weeks of LcS ingestion. A long-term study is needed to confirm the effects of LcS in HIV-infected children. Third, we used only bacterial 16S/23S rDNA concentrations in the plasma and bacterial 16S/23S rRNA molecules in the whole blood as metrics of microbial translocation, but did not use other biomarkers to evaluate the level of microbial translocation (e.g., lipopolysaccharide binding protein) due to the limited amount of the blood samples. Fourth, we could not include any kind of control group receiving a placebo (fermented milk without LcS) due to the difficulties in the preparation of the placebo, and in the recruitment of the age- and gender-matched children with and without HIV infection, especially those without ART.

In conclusion, our present results show that the ingestion of LcS-containing fermented milk induced an increase of CD4^+^ T cells, especially the Th17 subset, in both the HIV(+) and ART(+) groups without severe adverse events. LcS ingestion also induced decreases in plasma HIV load and CD8^+^ T-cell activation, and increases in total cholesterol and fasting blood sugar in the HIV(+) group, and decreases of AST and ALT in the HIV(+) and ART(+) groups, respectively. These findings suggest that short-term LcS ingestion may be a clinically safe supportive approach, with immunological, virological, and nutritional benefits in HIV-infected children.

## 4. Materials and Methods

### 4.1. Study Design and Subjects

We designed a nonrandomized, open-labeled, prospective study that included 60 Vietnamese children with vertical HIV infection. The recruitment of these participants was previously described [17]. Of the HIV-infected children, 31 had not received ART, because they did not meet the criteria to initiate ART based on the Vietnam guidelines for HIV/acquired immune deficiency syndrome (AIDS) Diagnosis and Treatment 2011 (HIV(+); 14 female, 17 male; median age, 6.2 years; range, 2.0–11.0 years); and 29 had received ART (ART(+); 12 female, 17 male; median age, 6.1 years; range, 3.6–8.6 years; median duration of ART, 3.5 years; range, 0.8–5.8 years). For these HIV-infected groups, the study inclusion criteria were as follows: (1) treatment/follow-up at the National Hospital of Pediatrics in Hanoi, Vietnam (NHP), (2) over 2 years of age, (3) no signs of AIDS, (4) no treatment within the prior 8 weeks that might influence the immune system, (5) no symptoms of gastrointestinal infections at the time of recruitment, and (6) no allergy to milk. The children in the ART(+) group resided at an orphanage center near Hanoi. The children in the HIV(+) group were followed at the outpatient department of NHP. This study also included a control group (HIV(−)) that comprised 20 healthy Vietnamese children without HIV infections (8 female, 12 male; median age, 4.1 years; range 2.0–8.3 years) who resided at another orphanage center near Hanoi. We tried to recruit all the children who met the inclusion criteria in this study.

### 4.2. Study Schedule

This study was carried out between May and August of 2012. The tested probiotic was a fermented milk containing *Lactobacillus casei* strain Shirota (LcS, Yakult Vietnam Co., LTD., Ho Chi Minh City, Vietnam). The fermented milk comprised 6.5 × 10^9^ colony forming units (cfu) of LcS, 51.7 kcal energy, 0.8 g protein, <0.1 g lipid, and 12.4 g carbohydrate in a volume of 65 mL per bottle. Each child ingested one bottle of the LcS-containing fermented milk every day for 8 weeks. We collected blood samples and clinical data from the HIV(+) and ART(+) groups at baseline (week 0), after 4 and 8 weeks of LcS ingestion (week 4 and week 8), and at 4 weeks after LcS cessation (week 12). For the HIV(−) group, samples and clinical data were collected at baseline (week 0) and after 8 weeks of LcS ingestion (week 8). Caretakers observed the children daily for clinical symptoms and LcS ingestion during the study period.

### 4.3. Clinical Laboratory Measurement

At the clinical laboratory of NHP in Hanoi, Vietnam, blood samples were analyzed for white blood cell (WBC) counts, WBC differentiation, hemoglobin levels, platelet counts (Beckman Coulter, Lh 780, Brea, CA, USA), liver function tests (AST/ALT), total cholesterol levels, and fasting blood sugar levels (Olympus AU640, Tokyo, Japan).

### 4.4. Plasma HIV Viral Load

Plasma HIV viral load (VL) was measured using the Cobas Taqman HIV-1 Test kit version 1.0 (Roche Molecular Systems, Inc., Branchburg, NJ, USA) following the manufacturer’s instructions as previously reported [17]. The detection limit for this kit was 40 copies/mL, and the plasma samples were diluted to 1:5.5 for these measurements, resulting in a final detection limit of 220 copies/mL.

### 4.5. Immunological Analysis

Immunological investigations were performed as previously reported [17]. Briefly, whole blood samples were stained with a combination of monoclonal antibodies to detect cell surface molecules. We used anti-CD4/CD38/HLA-DR and anti-CD8/CD38/HLA-DR antibodies as immune activation markers for CD4^+^ and CD8^+^ cells, respectively [27,28], and we used anti-CXCR3/CCR6/CD4 antibodies to define CD4^+^ cell subsets. CXCR3^+^CCR6^−^CD4^+^ cells were defined as Th1 cells, CXCR3^−^CCR6^−^CD4^+^ cells as Th2 cells, and CXCR3^−^CCR6^+^CD4^+^ cells as Th17 cells. CD25^high^CD4^+^ cells were defined as regulatory T (Treg) cells [29]. The plasma soluble CD14 (sCD14) concentration was measured using a human sCD14 Immunoassay kit (R&D Systems, Minneapolis, MN, USA), following the manufacturer’s instructions.

### 4.6. Detection of Bacterial Ribosomal RNA Genes (rDNA) in Plasma

Bacterial 16S/23S rDNA in plasma was quantified by quantitative PCR (qPCR) using the TaKaRa Taq kit (TaKaRa Bio Inc., Shiga, Japan) as previously reported [17]. The lower detection limit for the targeted gene was two copies/µL plasma.

### 4.7. Detection of Bacterial Ribosomal RNA Molecules (rRNA) in Whole Blood

Live bacterial 16S/23S rRNA in whole blood samples was quantified by reverse transcription-quantitative PCR (RT-qPCR) as previously reported [17]. The lower detection limit for the targeted bacteria was four cells/mL of blood.

### 4.8. Statistical Analysis

We used the Wilcoxon signed ranks test to analyze the changes of immunological status, physical status, and biochemistry status after LcS ingestion in each group. We used the Chi square test or Fisher’s exact test to compare the detection frequency of bacterial rRNA in whole blood samples, and of rDNA in plasma, before versus after LcS ingestion in each group. All analyses were performed using SPSS version 19 (IBMSPSS statistics 19, IBM Corporation, Armonk, NY, USA). A *p* value of below 0.05 was considered statistically significant.

### 4.9. Study Approval

This study was designed in accordance with the World Medical Association Declaration of Helsinki, the Japanese Ethics Guidelines for Human Genome/Gene Analysis Research, and the Vietnamese Ethics Guidelines. The protocol was approved by the Ethics Committees of Kanazawa University, Japan, and the NHP, Hanoi, Vietnam. For all participants, we obtained written informed consent from the family and/or guardian. This study is registered as UMIN-CTR: UMIN000015044.

## Figures and Tables

**Table 1:** Detection of 16S/23S rDNA in plasma before and after LcS ingestion and detected frequency.

Target Bacteria	HIV(+) (*n* = 31)				ART(+) (*n* = 29)				HIV(−) (*n* = 20)	
	Week 0	Week 4	Week 8	Week 12	Week 0	Week 4	Week 8	Week 12	Week 0	Week 8
*Clostridium coccoides* group	ND	ND	ND	ND	ND	ND	ND	ND	ND	ND
*Clostridium leptum* subgroup	ND	ND	ND	ND	ND	ND	ND	ND	ND	ND
*Bacteroides fragilis* group	ND	ND	ND	ND	ND	ND	ND	ND	ND	ND
*Bifidobacterium*	ND	ND	ND	ND	ND	ND	ND	ND	ND	ND
*Atopobium* cluster	ND	ND	ND	ND	ND	ND	ND	ND	ND	ND
*Prevotella*	ND	ND	ND	ND	ND	37.1 [1/29]	ND	ND	ND	ND
*Enterobacteriaceae*	ND	ND	2.0 [1/31]	ND	ND	ND	ND	ND	ND	ND
*Streptococcus*	17.8 [1/31]	13.8 [2/31]	1.8 ± 0.5 [3/31]	ND	ND	23.7 [2/29]	20.7 [1/29]	3.0 [2/29]	ND	3.5 [2/20]
*Enterococcus*	ND	ND	ND	ND	ND	ND	ND	ND	ND	ND
*Staphylococcus*	* 35.5 ± 15.0 # [7/31]	38.7 [2/31]	25.6 ± 6.5 [4/31]	19.8 ± 11.9 [9/30]	ND	26.4 [1/29]	49.1 [2/29]	16.2 ± 7.8 [5/29]	23.2 [1/20]	35.1 ± 43.8 [4/20]
*Pseudomonas*	2.9 [1/31]	ND	ND	ND	ND	1.9 [2/29]	1.5 [1/29]	ND	3.3 [2/20]	ND
*Lactobacillus casei* subgroup	ND	ND	ND	ND	ND	ND	ND	ND	ND	ND

The bacterial rDNA detection rates at weeks 4, 8, and 12 were compared with the rates at week 0 for each group using the Chi-square or Fisher’s exact probability test. No significant difference was found, all *p* values were > 0.05. ND: not detected (lower detection limit, two copies/µL plasma). *: mean copy numbers ± SD of detected subjects. #: frequency [detected subjects/tested subjects].
